# Linkage analysis using principal components of gene expression data

**DOI:** 10.1186/1753-6561-1-s1-s79

**Published:** 2007-12-18

**Authors:** Elizabeth J Atkinson, Brooke L Fridley, Ellen L Goode, Shannon K McDonnell, Wen Liu-Mares, Kari G Rabe, Zhifu Sun, Susan L Slager, Mariza de Andrade

**Affiliations:** 1 Division of Biostatistics, Department of Health Sciences Research, Mayo Clinic College of Medicine, 200 1st Street SW, Rochester, Minnesota 55905, USA; 2 Division of Epidemiology, Department of Health Sciences Research, Mayo Clinic College of Medicine, 200 1st Street SW, Rochester, Minnesota 55905, USA

## Abstract

The goal of this paper is to investigate the effect of using principal components as a data reduction method for expression data in linkage analysis. We used 45 probes normalized using the Affymetrix Global Scaling that had evidence of high heritability to estimate the first 10 principal components (PC). A genome-wide linkage scan was performed on the 45 expression values and the 10 PCs using 2272 single-nucleotide polymorphisms. Our conclusions were: 1) PC analyses under-performed the single-probe analysis for known signals; 2) the PC that best reproduced the single-probe analysis was primarily composed of that probe; 3) no new signals were detected in the PC analysis; 4) no new pleiotropic effects were detected in the PC analysis.

## Background

There is great interest in understanding genetic factors related to variable expression of genes. Recently, several studies have shown the first evidence of heritability of mRNA between individuals [1-6]. By treating the expression phenotypes for each transcript (or probe) as a quantitative phenotype, a variance-components linkage analysis could be used [1-6]. The expectation is to detect linkage signals between the gene expression values and genomic regions. As pointed out by William et al. [6], several issues plague these studies, including the selection of informative expression values. Principal components (PC) is a dimension-reduction approach [7] and it has been shown to be a valuable tool in linkage analysis of correlated phenotypes [8]. Multivariate linkage analysis has been shown to be useful in identifying genomic regions with pleiotropic effect [9]. Given that the PC approach is another way to combine information from multiple phenotypes, it can be hypothesized that PC analysis might also be helpful in the identification of pleiotropic effects. Because of the large number of expression phenotypes in the Genetic Analysis Workshop 15 (GAW15) Problem 1 data set, we first selected the most genetically informative phenotypes based on those with high heritability estimates [10]. In this paper, we examine whether combinations of correlated expression phenotypes improved the linkage signals using PC and whether the PC results suggest new pleiotropic effects.

## Methods

### Data

The GAW15 Problem 1 CEPH (Centre d'Etude du Polymorphisme Humain) data consisted of 196 participants from 14 three-generation pedigrees with 14 individuals per family, 4 grandparents, 2 parents, and 8 offspring. Two hundred and seventy-six arrays including data on 3554 probe sets on the Affymetrix Human Focus Arrays were provided by GAW15. These probe sets had been selected as those with greatest inter-individual variability from a total of 8500 probe sets [2].

### Selection of phenotype subsets

To increase the number of informative phenotypes, we excluded genes whose expression had little variation (standard deviation ≤0.3) and low call rates (absent calls >90%) across samples; 3306 phenotypes (probe sets) remained. We further reduced the number by identifying those that were most likely to be genetic based on heritability estimates from a polygenic model, resulting in 45 phenotypes. Additional details of the selection process can be found in de Andrade et al. [10].

### Principal components

Principal-components (PC) analysis is a data reduction technique in which each component is a linear combination of the phenotypes, each PC describing as much variability of the phenotypes as possible [7]. Because the 45 phenotypes are on a common scale, the decompositions were made using the unscaled covariance matrix. The first ten PCs accounted for 84% of the variance in the 45 phenotypes; 14 components would have been required to explain 90% of the variance.

### Genetic data

For a subset of subjects, including founders, we observed a large number of missing genotypes. Recognizing that missing data can impact identity-by-descent (IBD) estimation [11] when there is linkage disequilibrium, we reduced the extent of linkage disequilibrium between single-nucleotide polymorphisms (SNPs) by removing SNPs with *r*^2 ^> 0.30 using ldSelect [12]. Of the 2756 markers provided by GAW15, 2272 remained (mean spacing 1.2 cM). We then removed 2205 Mendelian inconsistencies (0.5% of matings/genotypes) primarily by removing the conflicting offspring genotypes. Multipoint IBD (MIBD) sharing among pairs of relatives was calculated using SIMWALK2 [13].

### Quantitative trait linkage analysis

Prior to the linkage analysis, the 55 phenotypes (45 expression phenotype + 10 PCs) were normally transformed using the empirical normal quantile transformation [14], which has been shown to have increased power for variance-components analysis [15]. Variance-components linkage analyses were performed using the S-Plus/R library *multic *[16]. Sex was used as a covariate. We assessed the 55 phenotypes for evidence of linkage and considered "strong" linkage evidence as *p *< 10^-9^, which is comparable to Table 1 in Morley et al. [2], and "moderate" linkage evidence as *p *< 10^-4 ^for comparison with the single probe analyses. Finally, for the 45 expression phenotype models, we used a screening tool proposed by de Andrade et al. [17] to estimate bivariate linkage results. For those phenotypes that suggested strong bivariate linkage using the screening tool, bivariate linkage analysis was also performed [18].

**Table 1:** Expression phenotypes with the strongest agreement and evidence of linkage for the Morley et al. [2], single-probe, and principal-components analyses

			Morley et al. [2]	Single probe			Principal components			
					
Gene	Location	Probe	*p*-value	LOD	*p*-value	Position (cM)	PC	LOD	*p*-value	Position (cM)
CH13L2	1p13.3	213060.s.at	<10^-10^	11.6	<10^-12^	111.8	3	2.9	<10^-3^	112.2
ZP3	7q11	210910.at	<10^-9^	13	<10^-14^	75.6	4	2.9	<10^-3^	64.3
PSPHL	7p11	205048.s.at	<10^-10^	6.1	<10^-7^	64.3	4	2.9	<10^-3^	64.3
DDX17	22q13	208151x.at	<10^-9^	5.9	<10^-7^	43.4	5	1.2	<10^-2^	47.7
										
UGT2B17	4q13	207245.at	---------	8.3	<10^-9^	62.3	2	7.1	<10^-8^	63.9
LRAP	5q15	219759.at	---------	8.1	<10^-9^	99	4	5.6	<10^-6^	97.7
HLA-DQB1	6p21.3	209480.at	---------	9	<10^-10^	36	1	10.7	<10^-11^	34.2
HLA-DPB1	6p21.3	201137.s.at	---------	8.8	<10^-10^	31.6	3	3.8	<10^-4^	38.3

The column "LOD" represents the maximum LOD scores in the chromosome where the genes are located with its respective *p*-values and positions in cM. The principal components results represent values at the same relative region as those found using the two other methods (single probe, Morley et al. [2]), even though the PC results are not composed of a single probe. The column "PC" lists the principal component that was used to find the maximum LOD score. The four bottom probes met our *p*-value < 10^-9 ^criteria but did not appear on Morley's top 13 list. There were an additional 9 probes on Morley's list that were not in our top 45.

## Results

**Figure 1:**
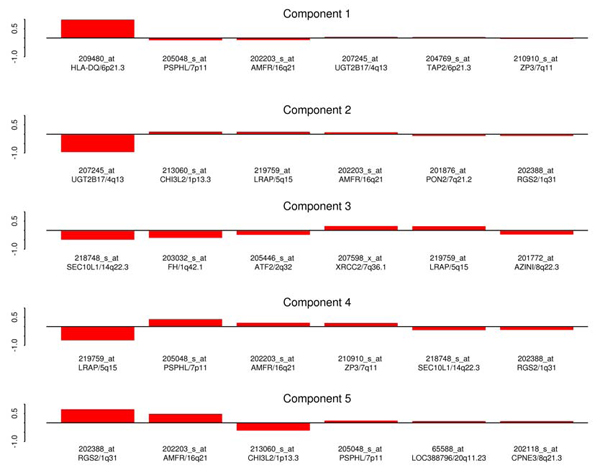
**Principal component loadings**. Loadings of the first five principal components along with the associated gene and chromosome location.

**Figure 2:**
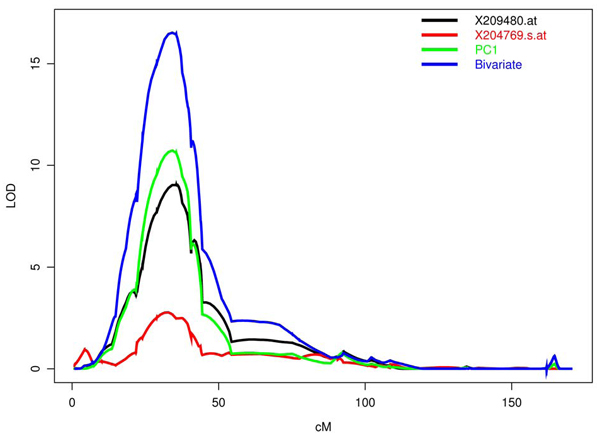
**Chromosome 6 linkage analysis**. Linkage results for chromosome 6 showing univariate analysis of two probes associated with the HLA region, the first principal component primarily composed of these two probes, and the bivariate analysis using these two probes.

Figure 1 shows the relative weighting for the first five PCs. For each component, the bar height represents the relative influence of a particular probe. Also included is the gene and gene location associated with each expression phenotype. The first PC is dominated by probe 209480.at, which has a relative weighting of 0.979. This first probe was the only component to show any increased linkage signal, changing from a LOD of 9.0 to 10.7. One possible reason for this increase is that the first PC is composed of two phenotypes (209480.at, 204769.s.at) that are associated with the HLA region on chromosome 6. Figure 2 shows the linkage analysis for chromosome 6 using these two phenotypes. Separate lines are drawn for the two univariate analyses, the first PC, and the bivariate analysis using these probes.

Table 1 compares our results with those of Morley et al. [2]. They used the Affymetrix normalization method and their multipoint genome-wide linkage analysis was done using SIBPAL in S.A.G.E. [2]. Two of our six best linkage signals for the single phenotype analysis agreed with Morley's; four, including the HLA region identified using the PC approach, were not found by Morley. For the remaining nine top phenotypes identified by Morley et al., we were unable to compare the results because the specific phenotypes were not part of our final 45.

Additional review of all the bivariate estimates using the de Andrade screening approach showed only two new areas to investigate that would not have been previously flagged using a criteria of *p *< 10^-9^. The *DDX17 *gene signal, identified by Morley using probe 208151.x.at (22q13.1), was increased from *p *< 10^-6 ^to *p *< 10^-8 ^when used with probe 207598.x.at (*XRCC2*, 7q36) in a bivariate analysis. One area in chromosome 6 had a screening *p*-value of 10^-8 ^using probes 220386.s.at and 320.at but actual bivariate analysis yielded a *p*-value similar to the stronger of the phenotypes (10^-6^). Neither of these potential associations were strongly grouped in the first 10 PCs. The Dead/H Box 17 (*DDX17*) is a member of the DEAD box (asp-glu-ala-asp/his) protein family of RNA helicases that are involved in diverse cellular functions including mRNA splicing, ribosome assembly, translation initiation, mRNA stability, and cell growth and division, and *XRCC2 *is essential for the efficient repair of DNA double-strand breaks by homologous recombination between sister chromatids.

## Discussion

We compared the total number of LOD scores greater than three across the genome for the first 10 PCs with the regions identified by each of the 45 phenotypes and found that in general, the PC analyses under-performed the single probe set analysis for known signals. The component that best reproduced the single probe set analysis (Component 1) was primarily composed of that probe set and also included another probe set that focused on that region. No new signals were detected using PCs despite the strong correlation between the probes. However, we observed a strong linkage signal on chromosome 6 in the HLA region when two probe sets from the HLA region were analyzed as bivariate traits. The two other increases in linkage signals from bivariate analysis that increased our list of interesting areas were not picked up using PCs.

## Conclusion

We observed that although PC has been suggested as a potentially useful screening tool for identifying genes linked to a cluster of highly correlated variables/phenotypes, it was not helpful in identifying linkage signals in this data set. Based on this analysis, other data reduction techniques should be investigated.

## Competing interests

The author(s) declare that they have no competing interests.
